# Barriers and Enablers Experienced by General Practitioners in Delivering Safe and Equitable Care during COVID-19: A Qualitative Investigation in Two Countries

**DOI:** 10.3390/healthcare11233009

**Published:** 2023-11-21

**Authors:** Esther Van Poel, Tessa van Loenen, Claire Collins, Kaatje Van Roy, Maria Van den Muijsenbergh, Sara Willems

**Affiliations:** 1 Department of Public Health and Primary Care, Ghent University, 9000 Ghent, Belgium; claire.collins@icgp.ie (C.C.); kaatje.vanroy@ugent.be (K.V.R.); sara.willems@ugent.be (S.W.); 2 Quality and Safety Ghent, Department of Public Health and Primary Care, Ghent University, 9000 Ghent, Belgium; 3 Radboud University Medical Centre, Radboud University, 6525 XZ Nijmegen, The Netherlands; tessa.vanloenen@radboudumc.nl (T.v.L.); maria.vandenmuijsenbergh@radboudumc.nl (M.V.d.M.); 4 Pharos—Dutch Center of Expertise on Health Disparities, 3511 MJ Utrecht, The Netherlands; 5 Research Centre, Irish College of General Practitioners, D02 XR68 Dublin, Ireland

**Keywords:** primary health care, general practice, equity in healthcare, vulnerable populations, community-oriented primary care, patient safety, quality of healthcare, COVID-19, infectious diseases, qualitative research

## Abstract

Addressing equity in healthcare is fundamental for delivering safe care to vulnerable patients, especially during COVID-19. This paper aims to identify barriers and enabling factors for general practitioners (GPs) in delivering safe and equitable care during the COVID-19 pandemic. Semi-structured interviews took place during May–July 2020 among 18 Flemish and 16 Dutch GPs. Thematic analysis of the interviews demonstrated that while GPs acknowledged a smooth information flow by governments and professional organizations on care guidelines, the fast-changing information challenged them to stay up to date. Media communication facilitated information dissemination but also fueled misinformation and miscommunication, creating unrealistic patient expectations. Certain guidelines and patient reluctance delayed necessary care. A shortage of personal protective equipment made GPs concerned about patient safety during face-to-face contacts. Teleconsultations became a popular alternative, but posed increased patient safety risks. GPs struggled to identify and reach vulnerable patients. Equitable care was hindered by time constraints; thus, having the appropriate materials facilitated such care. An interprofessional collaboration involving paramedical, social, and city services benefited patient safety and equity in healthcare. However, limitations in this collaboration pressured GPs. The unprecedented and resource-constrained environment challenged GPs’ capacity to provide the healthcare quality they aspired to deliver. A well-structured collaborative network involving all stakeholders could benefit safe and equitable care in future pandemics.

## 1. Introduction

The infectious nature of the SARS-CoV-2 virus highlighted the importance of patient safety during the COVID-19 pandemic. Patient safety is defined as preventing and reducing the risks, errors, and harm that occur to patients during care provision [1,2,3]. Within the framework of public health and social measures (PHSMs), there was a strong emphasis on prioritizing infection prevention and control as integral components of care processes and procedures. For example, increased attention was paid to hand hygiene [4], the triage of patients before they entered the practice, a limited number of patients in the waiting room, and adjusting procedures for repeat prescriptions to avoid patients attending the practice [5,6,7].

The global literature consistently shows that not only COVID-19 but also the PHSMs disproportionately affected vulnerable populations [8,9]. For example, people with an underlying health condition [10] and ethnic minorities [11] suffered greater mortality and morbidity when infected with COVID-19. Furthermore, people living in crowded and impoverished housing struggled to comply with quarantine and physical distancing measures, making them at higher risk for infection [12]. Subsequently, delivering safe care for vulnerable patients was challenging because such care required a more intense or different approach to comply with their unique needs [13,14].

Therefore, addressing equity was crucial when striving for patient safety. Delivering equitable care could effectively mitigate disparities in COVID-related health outcomes [15]. In addition, it might also enhance equitable access to essential resources and information, including personal protective equipment (PPE), testing, and vaccines [16,17]. Due to the usual long-lasting relationship with patients [18], general practitioners (GPs) were uniquely positioned to identify vulnerable individuals, monitor their wellbeing, and address the direct and indirect effects of the COVID-19 pandemic on their health by providing safe and equitable care.

Both patient safety and equity are integral domains of high-quality care [19]. This paper aims to identify barriers and enabling factors for GPs in delivering safe and equitable care during the COVID-19 pandemic. The results of this paper will capture cross-country lessons learned to enhance patient safety and equity in healthcare in general practice in the aftermath of COVID-19 and future pandemics.

## 2. Materials and Methods

### 2.1. Study Design

A qualitative research design was employed using semi-structured interviews among GPs in Flanders (Belgium) and The Netherlands. Mirroring the findings from two regions, which have similarities in terms of culture, language, demography, and healthcare needs but have distinct healthcare organizations and allocated responsibilities for GPs during COVID-19, contributes to developing cross-country insights to improve the preparedness for further pandemics. The research team had extensive expertise in qualitative research and healthcare quality in general practice. The consolidated criteria for reporting qualitative studies (COREQ) was utilized as shown in Supplementary Table S1 [20].

### 2.2. Study Setting

Belgium utilizes a mixed healthcare financing system, involving compulsory social health insurance funded by employers and employees, complemented by government support, private insurance, and out-of-pocket payments. Flanders is the Dutch-speaking northern region of Belgium and follows Belgium’s overall healthcare financing model. In The Netherlands, a mandatory health insurance system requires residents to obtain coverage from private insurers, with government subsidies ensuring accessibility and supplementary insurance available for additional services beyond the basic package. During COVID-19, Flemish GPs were actively involved in contact tracing, testing, staffing testing sites, and issuing sickness and quarantine absence certificates. GPs in The Netherlands were not involved in these activities.

### 2.3. Study Population and Data Collection

Between May and July 2020, 18 Flemish and 16 Dutch GPs participated in semi-structured interviews. In both regions, a purposive sampling approach was used to recruit and select GPs, ensuring diversity across various parameters, including gender, work experience, migration background, practice type, and location. All GPs were invited via email through the network of the Flemish and Dutch research teams. Notably, all Flemish GPs worked within a fee-for-service payment model, the prevailing model in Flanders. The payment system of the Dutch GP practices consists of a combination of fee-for-service, capitation, and pay for performance models. Due to the PHSMs in place at the time of the study, the interviews were conducted through an online video platform in Flanders, and over the telephone in The Netherlands. No repeat interviews were carried out.

Both in Flanders and The Netherlands, an interview guide based on expert opinion was used to guide the interviews. Although the two studies were initiated separately, their interview guides, while distinct, covered identical topics: (a) adaptation within the practice organization; (b) measures taken to ensure healthcare provision for vulnerable patients; (c) barriers and enablers in delivering this care; and (d) lessons learned. The Dutch interview guide placed more emphasis on the context of PHSMs, while the Flemish interview guide had a broader focus on the context of the COVID-19 pandemic as a whole. In both regions, the interviews lasted approximately 60 min. They were recorded and transcribed verbatim.

### 2.4. Data Analysis

The participating GPs were described based on their gender, years of work experience, migration background, and practice location. The Flemish transcripts were imported into NVivo release 1.6.1. and the Dutch transcripts into Atlas.ti. Thematic analysis was performed according to the six-step analysis procedure introduced by Braun and Clarke [21]: (i) familiarization with the data; (ii) generation of initial codes; (iii) searching for themes; (iv) reviewing themes; (v) refining and naming themes; and finally, (vi) producing the report. Steps i–iii were conducted by researchers E.V.P. and K.V.R. Initially, both researchers coded two interviews and subsequently convened to align on codes and emerging themes. M.V.d.M. then applied the preliminary Flemish coding structure to the Dutch interviews, enhancing and refining it. Multiple discussions between the Flemish (E.V.P.) and Dutch research teams (T.v.L., M.V.d.M.) led to the development of a shared coding tree. Subsequently, E.V.P. reevaluated previously coded interviews using the shared framework for the remaining interviews (see Table 1).

### 2.5. Trustworthiness of This Study

Data collection rigor was bolstered by recruiting participants with diversity across relevant factors until data saturation was reached, using an interview guide (while allowing adaptation to conversational flow), trained interviewers, and consistent coding. Data analysis involved discussions among the research team members, who possessed extensive experience in qualitative research in PC. In qualitative research, involving multiple researchers is recommended for enhanced data interpretation, as they can complement and challenge each other’s perspectives, enriching and refining the analysis [22,23].

### 2.6. Ethical Approval

The investigation in Flanders (project number BC-07695) was approved by the Research Ethics Committee of Ghent University Hospital, while ethics approval in The Netherlands was obtained from CMO Arnhem-Nijmegen (project number 2020-6428). The principles of the Declaration of Helsinki were followed. Informed consent was obtained from all participants.

## 3. Results

### 3.1. Characteristics of the Study Participants

Table 2 shows the characteristics of the GPs who participated in the interviews. The majority of the GPs in Flanders were male, (*n* = 11/18, 61.1%) whereas the majority in The Netherlands were female (*n* = 12/16, 75%). In both regions, the work experience of the participating GPs varied widely between 0 and 40 years. One Flemish GP and three Dutch GPs had a migration background.

### 3.2. Barriers and Enablers for GPs in Delivering Safe and Equitable Care during COVID-19

#### 3.2.1. Perceived Barriers and Enablers Related to the Patient

Both Flemish and Dutch GPs shared the experience that COVID-19 and PHSMs increased the demand for care and added to its complexity for numerous patients, particularly those already in a fragile state. GPs observed an increase in patients with anxiety problems, destabilization of formerly stable chronic diseases, psychiatric conditions, and increased reports of loneliness, stress, and depression. This increased vulnerability of patients underscored the importance of prioritizing patient safety to prevent and mitigate worsening conditions.

*“Debt is one of the biggest causes of stress. And stress is one of the biggest causes of disease”.*

*[GPN10]*

*“The psychiatric problems will rise; I already see it in some patients. Addictions will rise. These are the people hit by the first blows”.*

*[GPN9]*

In this context, timely access to healthcare services was important. Some patients disregarded safety guidelines when accessing healthcare and acted based on their personal preferences; others refrained from care and remained isolated in their homes for prolonged periods. Both attitudes and behaviors posed risks to patient safety, generating heightened concerns, especially for the latter group. According to the GPs interviewed, these patients frequently postponed seeking medical attention due to concerns about bothering the GP or getting infected with COVID-19, even for critical conditions that could lead to severe consequences.

*“The biggest danger are the people who haven’t been called. Those are the tragedies that we saw afterwards. […], a man who thought he had corona and didn’t call. And he ended up having an infarction”.*

*[GPF13]*

*“… fear among people anyway, fear of disturbing us, not daring to call… because they think we are too busy, or fear to come here, we see that with some older people, because they think they will get infected here in spite of all the measures, so they don’t dare to come anymore”.*

*[GPF2]*

Setting up initiatives to enhance access to healthcare for vulnerable patients could reduce patient safety incidents; however, being able to identify the patient’s vulnerability is a necessary prerequisite for this. GPs considered patients’ reluctance to disclose their vulnerabilities, such as financial difficulties or living in violent home situations, as a barrier to safe and equitable care.

*“If they say, you should send the bill of this consultation to my administrator, well then you know (…) But sometimes they have debts and you don’t know, or they don’t want to say anything about it. Or there is domestic violence and they don’t tell”.*

*[GPF3]*

*“But to me, vulnerable people are…, again in my kind of practice… kind of invisible… They’re not going to tell you”.*

*[GPF9]*

#### 3.2.2. Perceived Barriers and Enablers Related to the GP Practice Staff

To ensure patient safety in the unprecedented circumstances of the pandemic, GP practices were committed to revising their daily organization and the processes and procedures in the short term. In this regard, it was considered helpful to establish clear and shared agreements with the team. Some practices even decided to implement permanent changes to their organizational structure in response to the post-COVID-19 era.

*“We changed a lot in our practice, but in that way of, look, we’re probably not going to step back for most of those things”.*

*[GPF16]*

The available resources played a crucial role in this reorganization. Due to a lack of PPE, GPs could not visit or see their patients for some time. GPs were more worried about transmitting COVID-19 to patients than about being infected themselves.

*“You can’t go to your vulnerable population because you… yes, you are limited in your protective equipment. You can’t take risks… You can’t pass on the infection yourself of course”.*

*[GPF18]*

*“We had to be very careful with the personal protective equipment. […] There were shortages and occasionally, there were mouth masks, which were then rejected anyway”.*

*[GPN2]*

Teleconsultation appointments were widely adopted as an alternative to face-to-face consultations for patient care. Introducing video consultations was a new experience for many GPs, with only a few of them having previously experimented with this method. Most importantly, GPs often felt uncertain about making diagnoses and their actions during teleconsultations due to the lack of face-to-face contact or clinical examination.

*“But you do notice that those are just unpleasant circumstances to work in, especially because… Yes, a lot is handled by telephone. A lot of doubt of, have I, handled it properly now? Did I explain it sufficiently? Are those people going to listen to what I said now? Because yes, you only hear them on the phone. You can say: you are not allowed to go outside, they might go outside anyway. So… […], a lot of frustration, a lot of uncertainty”.*

*[GPF14]*

The GPs reported that, especially when dealing with new or vulnerable patients, teleconsultations increase the risk of misunderstandings as patients might face increased difficulties expressing their questions and concerns. As a result, GPs felt the need to place more time into providing patients with information and exploring their questions. Additionally, GPs encountered challenges in relying on their intuition to assess their patients’ health. Therefore, patient safety incidents such as a wrong or delayed diagnosis happened.

*“Most of the patients who call, we do know. We had a few that we really didn’t know. This was really difficult”.*

*[GPF12]*

*“We had a very unpleasant experience. A patient called, and her problem was handled by telephone, but it turned out to be a metastatic cervix carcinoma. That was very intense. It was the language barrier. Someone who couldn’t explain her symptoms properly and we miss that gut feeling by phone. We missed it completely”.*

*[GPN15]*

Overall, the workload during COVID-19 was experienced very differently. Some GPs expressed concerns about the quality of healthcare they delivered, often carrying work-related worries home with them. Working days often finished earlier, but the pandemic also put pressure on the mental and emotional resilience of GPs. The new working conditions, including permanent infection prevention and control awareness, made GPs feel more tired than in the pre-COVID era.

*“I think it is mainly heavy, because you’re constantly thinking about it. (Laughs) And because you suddenly have to consider safety where you normally wouldn’t have to. That reminds me of friends who worked in Kenya twenty years ago. They said: we spend half the day worrying about safety. And we’re not used to that, are we?”*

*[GPF7]*

*“I really enjoy crisis moments. Even though I felt tired, didn’t sleep well, which I normally don’t do, there was a lot of excitement and tension. So, I don’t care too much”.*

*[GPN7]*

Recognizing the vulnerability of patients, numerous GPs believed that ensuring patient safety in vulnerable patients required the adoption of an alternative approach. For example, GPs provided more time for vulnerable patients during consultations, used layman’s terms, and checked whether instructions were properly understood.

*“But we have to… take more account of that. So, for example, if we have a patient of a low social class on the phone, that we take more time for this. That we also ask those kind of control questions where we can check, did they understand it correctly. And to make them repeat certain things, so that you know, okay, yes they understood”.*

*[GPF13]*

The distribution of multilingual brochures was carried out to ensure effective communication with non-native-speaking patients. In this context, having appropriate materials readily available was seen as a facilitating factor.

*“At a certain point, we realized that our information in Dutch was insufficient for a small proportion of our patients. And then, we searched for French-language and English-language information, printed it, hung it out, both at the front door and in the waiting rooms, and we also gave it to them”.*

*[GPF5]*

Furthermore, the unprecedented circumstances of COVID-19 accelerated the adoption of innovations to enhance equity in healthcare. One such example is outreach work, where healthcare providers proactively engaged with vulnerable patients through telephone calls or by delivering information to their mailboxes to assess their health and living conditions.

*“… we did call some people on an individual basis as well. Proactively…asking: How are you doing? And do you have food? And, you live on the umpteenth [a high] floor, is there someone who occasionally comes to see you? Is there someone who calls once in a while? I mean, are you safe in general?”*

*[GPF10]*

*“Calling vulnerable families to ask, ‘how are you?’, I did that much more proactively during the lockdown than I did before”.*

*[GPN2]*

However, GPs experienced challenges in reaching out to vulnerable patients during COVID-19, which added pressure to delivering both safe and equitable care. The difficulty arose from various factors, including GPs lacking accurate contact information for some patients and encountering language barriers when attempting telephone communication.

*“Then you could only hope people took their phones. Our patients can suddenly have a different phone number because it is cheaper or for another reason”.*

*[GPN9]*

*“It’s not always obvious or even often you suddenly notice that the consultation has been cancelled. Then you say, we’ll call back, because he must come. Yes, then you end up straight to a voicemail of which you don’t understand anything. Or you end up with someone who doesn’t know what you’re talking about. So it’s not easy to reach them either. This also gave a feeling like, damn, we have to see them”.*

*[GPF12]*

Initiatives typically began with the identification of vulnerable patients. The participating GPs were convinced that an interprofessional practice team, which includes a social worker, could enhance this identification process.

*“[…] I have very little insight into patients’ financial situation, mainly also because there are no social workers here in the practice”.*

*[GPF4]*

The information in the electronic medical records was valuable in identifying patients with a chronic condition or financial problems. In Flanders, the latter was usually deduced from the patients’ reimbursement status. Also, the electronic medical record (EMR) offered the possibility to register other personal or social characteristics, but this was not performed systematically by GPs. It follows that GPs struggled to identify other vulnerable patients during COVID-19.

*“You can extract patients with diabetes from your medical files. But patients who are underprivileged are not marked in your file as ‘underprivileged’”.*

*[GPF15]*

Active questioning during patient consultations can also serve as a means of identification for vulnerability. Engaging in discussions about patient vulnerability was frequently challenging. Certain GPs hesitated to broach sensitive topics, such as financial issues.

*“Yeah, it’s a bit weird anyway to ask as a physician, ‘Are you still getting by financially at the end of the month?’ (Laughs)”.*

*[GPF14]*

In the context of patients with a known situation of domestic violence, GPs felt untrained to follow up through teleconsultations.

*“A very elderly mother living with her, yes, at times, violent daughter. Where we have already tried everything. And nothing works. Yes, now it certainly isn’t working out, is it? […] I’ve been postponing it. What should you do if the daughter picks up the phone?”.*

*[GPF7]*

Despite acknowledging the importance of equitable care, many GPs faced obstacles in organizing initiatives due to limited time.

*“Now it is actually for me still… trying to survive. […] It is only trying to help people as much as possible. But taking up extra things, like for example… which could be very useful… calling people… of whom you know that it has been a long time since they’ve been here, I’d like to know what’s wrong. That’s not happening at the moment. Nor can I. […] At the moment I am rather solving problems…”.*

*[GPF1]*

#### 3.2.3. Perceived Barriers and Enablers Related to Other Healthcare Professionals and City and Social Services

COVID-19 offered an opportunity to boost existing collaborations with other healthcare providers and social welfare services or to start new collaborations, enhancing both safe and equitable care. Flemish and Dutch GPs reported a strong sense of collegiality between GPs in their neighborhood to help each other. Live meetings were temporarily canceled, but online tools enabled GPs to stay in touch and help each other if needed.

*“In the beginning, I felt I had to do it all on my own. Until at one point, I asked for help from the other GPs to participate and they were all willing to do that, so I just should have done that earlier”.*

*[GPN1]*

Also, GPs expressed appreciation for medical specialists who were available for advice. In many cases, COVID-19 led to enhanced collaboration between primary care (PC) and secondary care. Some GPs expressed that COVID-19 united them.

*“I thought there was… really good contact with the physicians from secondary care. They were really accessible; they were really open to giving advice and so on. I thought… in terms of cooperation between primary and secondary care, I thought it was very good”.*

*[GPF2]*

However, in cases where this collaboration did not take root, GPs felt frustrated because patient follow-up was compromised.

*“I was told by a psychiatrist ‘We can’t see anybody for the time being.’ And I thought ‘Hellooo, I am doing it too, you know. Why can’t a psychiatrist do a consultation at 1.5 m distance?”*

*[GPN5]*

*“Suddenly, the poor GP is a bit on his own with the problems we can’t solve. Then, we have to make do with what we have and that is limited […] I think it’s weak that such a system is unable to provide continuity at the slightest”.*

*[GPF12]*

GPs described their fear for the health of those patients whose non-urgent consultations and interventions in the hospital were postponed. Sometimes, alternative consultation forms were introduced; however, many GPs voiced concerns about their limited accessibility to certain vulnerable groups. GPs often reported taking over the care where they could mitigate additional health risks. This contributed to increased feelings of responsibility among GPs.

*“I treated some people with heart failure myself, because the cardiologist only held telephone consultations, which won’t work for this population [referring to vulnerable patients]”.*

*[GPN11]*

Also, limited personal availability of professionals working in city, social, and paramedical services was considered risky for patient health.

*“I found it very inconvenient that the physiotherapists did not work, people had started working from home and couldn’t continue or got overloaded”.*

*[GPN7]*

*“Now I am going to be blunt. I think the way the mental health care and the process of providing allocating homecare were paralyzed is unacceptable. Organization XX canceled their home visits, Organization YY still hasn’t restarted yet. That just cannot be!”*

*[GPN11]*

In addition, GPs considered city stakeholders as important partners in enhancing patient safety and equity in health. For instance, they recognized the importance of working with city stakeholders to develop appropriate communication materials to reach and properly inform vulnerable patients about COVID-19 and PHSMs.

*“I don’t think so, but then maybe it should be explored whether from the city… yes, because who else should provide objective information… objective or clear information can be provided to those groups at their own level and in their own language. Literally and figuratively”.*

*[GPF12]*

Often, the availability depended on benevolence at the individual or organizational level rather than being well-coordinated and organized. In the case when this collaboration was expanded, this allowed GPs to monitor the health and welfare of their patients, despite a lack of personal contact with the patient.

*“Now another thing is… my colleague has deliberately called a number of people with severe psychiatric disorders. Yes, and then, when you have them, you see that it is even more difficult. It was great that the mobile crisis team continued to work. But that depends on who led it. Because it wasn’t the case everywhere”.*

*[GPF7]*

Moreover, many GPs reported taking up a more coordinating role in care, whereas the more practical implementation of procedures was delegated to other healthcare providers, such as home care nurses. Effective collaboration was closely intertwined with clear agreements and transparent communication.

*“I sometimes had the feeling of being able to confine myself more to the essence of my job […] You have a home nursing team and they do a home visit and they catch problems. And those problems… They filter the problems. They identify those problems. […] And they did or applied what I thought had to be done, including wound care, follow up of antibiotics. […] That’s how I think healthcare should be organized. That we as physicians do the intellectual act, the coordination of this and that should be done”.*

*[GPF9]*

In cases where a strong interprofessional collaboration for vulnerable populations existed before COVID-19 was implemented, GPs experienced that they could find each other more easily during COVID-19 to set up initiatives. An example includes neighborhoods in The Netherlands where ‘Krachtige Basiszorg’ (powerful basic care) had been implemented. This approach aimed to enhance PC services in vulnerable neighborhoods by fostering collaboration between healthcare professionals, integrating care across disciplines, and empowering patients in their healthcare decisions.

#### 3.2.4. Perceived Barriers and Enablers Related to the Government and Professional Organizations

Both in Flanders and The Netherlands, governments and professional organizations developed and distributed the PSHMs, including the guidelines for organizing safe GP care during COVID-19. GPs experienced the information flow as smooth, but continuous updates of the guidelines, often weekly or sometimes even daily, made it challenging to keep track.

*“But I actually feel it is quite possible to stay up-to-date. It’s a lot of reading, but it’s there”.*

*[GPN2]*

*“Because everything that was true today, no longer is true tomorrow. Everything that was obliged today, is not allowed tomorrow”.*

*[GPF10]*

Some GPs were confused when guidelines were contrary to the pre-COVID era guidelines. For example, using a regular email to provide patients with documents was allowed again, which contradicted the General Data Protection Regulation (GDPR) implemented in 2018.

*“Regarding privacy… all that turned out not to apply anymore. Just send it via Gmail, Hotmail, all kinds of emails. It didn’t have to be sent via secure channels anymore. Then, I also ask myself, why have we been forced into all kinds of things for two years?”.*

*[GPF12]*

Moreover, not all GPs experienced the guidelines and procedures as realistic to implement in their contacts with patients or the practice organization. Particularly, the compulsory temporary closure of GP practices was considered problematic. In The Netherlands, GPs were encouraged to work from home if possible. This led some patients to believe GP care was not available at all.

*“To my great disappointment, because to my feeling yes… A patient calls, because he is not feeling well and in these circumstances the GP, the doctor is supposed to say… I don’t want to see you, I am not allowed to see you. I do not want or am not allowed to come to you. Which in my view is a total mockery of what good general practice is. […]. You are afraid of me. That’s the biggest disillusionment in my whole career… […] I find it all so ambivalent and so easily said from behind your desk somewhere in Brussels or God knows where they decide it”.*

*[GPF12]*

*“There is a lot of overdue care, I think. People who called 3–4 days after a stroke because they didn’t want to burden us […]”.*

*[GPN11]*

Not only were GP practices temporarily closed, but the government also introduced a temporary ban on non-urgent care in hospital care to safeguard the capacity to care for COVID patients. However, this measure led to delayed diagnosis and treatment among non-COVID patients with urgent conditions.

*“‘We have had patients…, yes, collateral damage again. Patients who had pain in the legs. Which I didn’t trust from the beginning, and where I said, I don’t put a diagnosis on it here, but I know what examinations I want. But I called the hospital and it was, sorry colleague. We would like to see patients, but we are not allowed by the management. […] Well, the management followed the instructions of the government. No consultations, because this could result in admissions and we have to avoid admissions now, because we have to keep the beds free for Corona patients”.*

*[GPF6]*

In addition, Flemish GPs were given additional responsibilities during COVID-19, such as contact tracing, which involves identifying, evaluating, and managing individuals who have been exposed to COVID-19, aiming to monitor viral transmissions and perform timely interventions. Nevertheless, some GPs were hesitant about embracing this role, as it could potentially jeopardize the trust they had established with their patients.

*“With the triage and the track and tracing… I also don’t know if we are in a good position for this. We are confidential counsellors, you know. And we suddenly have to put people at home for two weeks. Uh, none of this is self-evident”.*

*[GPF7]*

Some GPs indicated that the fragmentation of care and responsibilities of the different authorities in Belgium caused problematic situations, such as the deficient provision of PPE and poor care delivery in residential care centers.

*“Only you see that the support of primary care regarding mouth masks, safety equipment and so on… that this was a, a…a drama. But then, then you see that care… and residential care centers are a clear example of that, in this situation where responsibilities are spread across the regional and federal level. Then it falls short. Yes. So the, the organization of care and organization of the disease. Disease is a federal matter and care is then mainly Flemish matter, this actually goes wrong”.*

*[GPF4]*

Moreover, a Flemish GP compared the assigned roles of GPs during COVID-19 to the organization of care in The Netherlands, suggesting that certain aspects could be improved by adopting the Dutch approach. An example includes the writing of certificates, which is managed by a separate organization in The Netherlands, unlike in Flanders, where it is the responsibility of GPs.

*“There are also tasks that went to the GP of which I think, I don’t know if it’s good. Also the certificates that have come to us. I think that it would be much better if it was organized like in The Netherlands, that a separate organization… In The Netherlands the GGD does that (referring to the municipal health services)”.*

*[GPF7]*

#### 3.2.5. Perceived Barriers and Enablers Related to the Media

Campaigns and information provided through the media could facilitate patient safety as they drew attention to COVID-19 and PHSMs by making it central to their coverage for weeks. However, the sensational reports about COVID-19 spooked many people. GPs believed that this resulted in healthcare providers dropping out of service.

*“Because in the media in recent weeks has been about little else but that. The images you see are images of care and so on. So these are images that… that instill fear”.*

*[GPF4]*

*“Who did fall out easily, especially in the beginning, those were the caregivers. […]. The media played a tremendously bad role in that. I’m convinced of that. They highlighted the need for mouth masks in such a way and highlighted the shortage of mouth masks in such a way that everyone thought that from the moment they were going to meet someone or had to go to work themselves without a mouth mask, that they were a goner anyway”.*

*[GPF6]*

Thus, some concerns here include the importance of accurate information and emphasis. For example, there was a lot of misinformation about COVID-19 testing. As a result, GPs were inundated with patients requesting testing, but available resources did not allow it, leaving many patients displeased.

*“It’s also sometimes difficult for us to see from… the, the ‘case’ definition of possibly COVID-19 changed daily and even now every few days and then the question of okay, who should be tested and who should not be tested, that’s also constantly evolving. And, and sometimes contrary to what that is then in the newspaper. So what you sometimes get is a discussion about, I want to be tested and we say, yes but you don’t meet the criteria to be tested”. […].*

*[GPF4]*

Furthermore, GPs felt that traditional media channels did not reach vulnerable patients, such as people with limited knowledge of the local language, leading to heightened anxiety and a lack of crucial information regarding COVID-19.

*“Yes, with the non-native speakers, the average information was… yes, very… much harder. Everyone who watches the news and who speaks Dutch, who was… who got information fed up”.*

*[GPF15]*

*“They go to the supermarket in complete protection suits and preferably don’t go outside the house at all. Sometimes I think the fear and irrationality is more present than indifference”.*

*[GPN4]*

## 4. Discussion

### 4.1. Main Findings

The results demonstrate that GPs recognized the significance of patient safety and equity in healthcare. However, the exceptional circumstances and resource-restricted environment tested their capacity to deliver the quality of care they aspired to provide.

One of the enablers in delivering safe care among Flemish and Dutch GPs was a seamless information flow on PHSMs from government and professional organizations. However, this finding contradicted the results of the cross-sectional PRICOV-19 study in The Netherlands [24]. More precisely, the Dutch PRICOV-19 study data showed that most practices relied on information from the independent research body, the ‘National Institute for Public Health and the Environment’, and professional organizations, instead of the government [25]. Moreover, the GPs from this study experienced challenges in staying up to date on PHSMs because of the rapidly changing care guidelines. This finding aligned with the existing evidence, as about half of the Belgian and one-third of the Dutch GPs indicated allocating sufficient protected time in GPs’ schedules to stay up to date with guidelines during COVID-19 [26]. Hoernke et al. [27] showed earlier that this situation might lead to confusion and distrust among GPs. This was illustrated in this study by the confusion regarding the use of a regular email to provide documents to patients.

To support patient safety in PC, guidelines on care provision should be robust and comprehensive [28]. While clear practice agreements for implementation within the practice were perceived as helpful in this study, the interviewed GPs believed that not all PHSMs could be integrated into their organization. Particularly, the compulsory temporary closure of GP practices raised concerns due to the increased pressure on accessibility, posing a threat to patient safety as PC is the first point of access to healthcare. When practices reopened, GPs and patients were obliged to wear face masks during face-to-face consultations. However, there was a PPE shortage at the same time which prevented consultations.

During COVID-19, teleconsultations became the norm in GP practices to conduct remote patient visits as an alternative to face-to-face consultations. Nevertheless, the interviewed Flemish and Dutch GPs perceived an increased risk of patient safety incidents due to their uncertainty in relying on intuition for diagnosis and the absence of clinical examination and options to verify patients’ understanding of instructions, especially among unknown or vulnerable patients. This anxiety is confirmed in other research, demonstrating that about one-fourth of European practices faced a delayed care process in a patient with an urgent condition due to a misjudgment during a telephone triage [26].

Next, the broad reach of media communication was beneficial for patient safety in informing the general population about the evolution of COVID-19 and instructing on PHSMs. However, misinformation and miscommunication on social and traditional media platforms resulted in unrealistic expectations and fear among patients. For example, Flemish GPs encountered a surge in patient requests for testing, despite limited resources and policies restricting widespread testing. This highlights the adverse influence of misinformation and miscommunication with regard to patient safety, which aligns with previous evidence and may contribute to vaccine hesitancy [29,30].

The confusion around communication left the interviewed GPs feeling that some PHSMs and patient reluctance led to care postponement with sometimes devastating consequences. This confirmed the findings of earlier studies, demonstrating that most Belgian and Dutch practices faced a delayed care process in patients with urgent conditions [25,31]. The concerns of the interviewed GPs also aligned with the previous research that indicated high rates of unmet healthcare needs in patients during COVID-19 [32].

Another barrier to safe care in Flanders was the fragmentation of care and responsibilities of the different authorities in Belgium, which hindered an effective policy and the provision of PPE. Regarding policymaking, multiple studies reported that GPs felt sidelined during COVID-19 [33,34]. In this study, a few Flemish GPs expressed uncertainty about their role in COVID-19. In contrast, Dutch GPs were not engaged in contact tracing, testing, staffing testing sites, or issuing sickness and quarantine absence certificates. Also, all interviewed GPs expressed that the novel working conditions, involving constant concerns about infection prevention and control, strained their resilience. A 2022 systemic review on the wellbeing of GPs during the COVID-19 pandemic reported that numerous GPs suffered from reduced work satisfaction, anxiety, symptoms of depression or burnout, and physical symptoms during COVID-19 [35]. Poor wellbeing among those working in PC has been linked to patient safety issues [36,37].

Equitable care in general practice is crucial due to the persistence of the ‘inverse care law’, where patients in greater need of healthcare often have less access, while those with lesser healthcare needs have greater access to medical services [38]. The interviewed GPs were strongly aware of patient vulnerability and were devoted to organizing initiatives for vulnerable patients. Hence, the availability of multilingual communication materials facilitated these efforts. Earlier research in the pre-COVID era demonstrated its significance, as addressing language barriers in patients with limited knowledge of the local language reduced the risk of comprehension problems or confusion with instructions [39]. Despite the GPs’ dedication, this study’s results showed that limited time was a major barrier for setting up equitable care, which is confirmed in prior studies [40,41]. Similarly, Bouchez et al. [42] highlighted that the practice type and personal characteristics of GPs also played a determining role in commencing initiatives during COVID-19.

Both Flemish and Dutch GPs found identifying and reaching vulnerable patients, which they considered as prerequisites for establishing equitable care, challenging. Flemish GPs primarily used the EMR for identifying patients with chronic conditions or financial problems, as registering personal or social characteristics in the EMR was infrequent. Another barrier in this study to delivering equitable care was the reluctance to discuss patient vulnerabilities. On the one hand, GPs felt that patients occasionally hid their vulnerabilities, which is consistent with the existing evidence, where victims of domestic violence often take multiple visits to disclose abuse due to fears about their safety and potential consequences [43,44]. On the other hand, some interviewed GPs hesitated to broach these sensitive topics during consultations or felt untrained to address difficult situations. However, in the case of domestic violence, directly asking victims about abuse facilitated disclosure to the healthcare provider [44]. Furthermore, the literature is inconsistent regarding GPs’ awareness and knowledge of the overall social factors contributing to patients’ health issues [45,46,47].

According to GPs, being an interprofessional practice team enabled equity in healthcare. The significance of adopting such a team approach in caring for vulnerable patients is broadly recognized [48]. In this study, GPs perceived that a strong collaborative network with other healthcare providers, social providers, and city services benefited both the safety and equity of patients in healthcare during COVID-19. This collaborative framework facilitated safe patient monitoring and enabled holistic initiatives for vulnerable patients. Notably, this often involved role adjustment, with GPs assuming coordinating roles while others managed practical aspects. GPs also reported an enhanced collaboration with medical specialists, which is consistent with prior evidence [49]. However, they observed that the availability and accessibility of healthcare and city services were more dependent on individual or organizational goodwill rather than coordinated planning. Their communication with GP staff was frequently deficient. Nonetheless, Salas et al. [50] underlined that effective teamwork hinges on clear communication.

### 4.2. Implications for Policy, Practice, Training, and Research

According to European research, GPs expressed low levels of preparedness in dealing with the COVID-19 pandemic [51]. This is problematic considering GPs’ key role during COVID-19 as well as previous pandemics [24,52]. International comparisons underscore the need for improved collaboration between PC and other sectors to respond to pandemics effectively [53]. Using an intersectoral approach, this study offers valuable insights for preparing GPs to enhance the provision of safe and equitable care in the aftermath of COVID-19 and in future pandemics.

First, this study emphasizes the importance of involving GPs in policymaking at the highest level to consider their feedback and insights from their COVID-19 experience. On the European level, cross-country exchange of insights can enhance readiness for future pandemics [52,54]. A cross-country collaboration can facilitate the optimal use of available healthcare resources, including facilities, personnel, equipment, technologies, and funding.

To address the concern of GPs feeling excluded, engaging them in defining their role in ensuring patient safety and equity in future pandemics is crucial as a first step forward. Inspiration can be drawn from Canadian research, which explicitly described GPs’ roles for all five distinct stages of a pandemic response [55]. The uncertain evolution of the pandemic and the continuous accumulation of evidence constituted to the rapid change of guidelines during COVID-19 being inevitable [56]. To facilitate knowledge revision on PHSMs among healthcare providers, international experts suggested acknowledging the uncertainties and emphasizing the conflict between the old and new information in communication [57,58]. Active participation of GPs in developing PHSMs is essential for practical and applicable care guidelines. Also, a comprehensive strategy involving the media sector, governments, academics, healthcare providers, and the public is necessary to combat misinformation and miscommunication. Given that traditional media may not effectively reach vulnerable patients, collaboration with city services can help tailor media content to local community needs.

Other important stakeholders in delivering safe and equitable care are the educational institutions, as they play a role in training current and future GPs to enhance healthcare quality and bolster workforce resilience. With the ongoing digitalization of healthcare, it becomes imperative to provide training to equip (future) GPs with the necessary skills for conducting secure remote consultations, particularly with an emphasis on addressing the needs of vulnerable patients.

Next, GP practices should foster open discussions among staff about their organization and healthcare provision during COVID-19, emphasizing patient safety and health equity as initial steps for quality improvement projects. Drawing from this study, safety-focused initiatives could target enhancing practice accessibility to avoid care postponement. In terms of equity in healthcare, opportunities for enhancement lie in systematically questioning patients’ personal and social characteristics and documenting these in the EMR, which could enhance the identification of vulnerable patients. Engaging in learning networks facilitated by professional organizations could provide GPs with a valuable platform to exchange their good practices in organizing initiatives.

Finally, the World Health Organization has advocated patient participation as a key strategy to enhance healthcare quality and outcomes. Therefore, future research should prioritize investigating patient perceptions regarding the delivery of safe and equitable care in general practice during COVID-19 and their role in enhancing it for the future. Collecting the personal and social characteristics of patients is crucial for designing targeted interventions based on the findings.

### 4.3. Strengths and Limitations

To the best of our knowledge, this paper is the first to explore the barriers and facilitators for GPs in delivering safe and equitable care during COVID-19 through a qualitative interview study. This study’s findings enhance the depth of comprehension in this field, complementing the predominantly quantitative studies conducted thus far. The triangulation of the Flemish and Dutch data contributed to developing cross-country insights and resulted in an in-depth understanding of this topic. However, a few limitations should be noted.

First of all, participation was voluntary; thus, a selection bias cannot be ruled out. Purposive sampling was used for recruitment to gather opinions from GPs working in different settings. Data collection continued until saturation was achieved. However, the notion of ‘vulnerable patients’ refers to many factors, including sociodemographic and socioeconomic attributes, underlying health conditions, healthcare accessibility, and other elements. Considering the dynamic nature of the COVID-19 pandemic and the increasing knowledge about its effects on vulnerable patients, it is possible that this evolution could have an impact on the perceived barriers and enablers for GPs. Moreover, this study was conducted in two countries, implying that confounding factors related to the differences between both countries cannot be excluded. We also did not collect data on the intensity of the barriers or enablers experienced. Another limitation is that none of the interview transcripts were returned to the participating GPs for comments or corrections.

In addition, the data were collected in the early phases of the COVID-19 pandemic. Given the dynamic nature of the COVID-19 response, which demanded frequent short-term adjustments in general practice, the factors influencing safe and equitable care delivery for GPs might have evolved throughout the pandemic. Conducting longitudinal studies to validate these findings would provide more conclusive insights.

Finally, the validation of qualitative research is constrained by inherent subjectivity and the context-bound nature of the findings. However, the involvement of multiple researchers in evaluating and analyzing transcribed interviews enhanced the reliability of the findings.

## 5. Conclusions

The interviewed Flemish and Dutch GPs were strongly committed to enhancing patient safety and equity in healthcare during COVID-19. Nevertheless, the unprecedented circumstances and resource-limited environment challenged their ability to provide the quality of care they aspired to deliver. A close and well-organized collaboration between all stakeholders could benefit safe and equitable care in the aftermath of COVID-19 and future pandemics.

## Figures and Tables

**Table 1:** Barriers and enablers for GPs regarding delivering safe and equitable care linked to different stakeholders.

	Delivering Safe Care		Delivering Equitable Care	
	Barrier	Enabler	Barrier	Enabler
Patient	- Increased care needs - Reluctance to care contacts - Non-compliance with PHSMs - Lack of openness about own vulnerabilities	/	- Lack of openness about own vulnerabilities	/
GP practice staff	- Unfamiliarity with the ‘new’ working conditions - Shortage of personal protective equipment - Treatment of unknown or vulnerable patients - Lack of training in teleconsultations - High workload and pressure on own health	- Commitment to comply with PHSMs and deliver safe care - Clear and shared agreements within the practice - Awareness of patient vulnerability	- Shortage of time - Reluctance to discuss patient vulnerabilities - Lack of systematic registration of personal and social characteristics of patients in the electronic medical record - Difficult identification and contact with vulnerable patients - Lack of training in addressing difficult situations	- Awareness of patient vulnerability - Commitment to set up initiatives for vulnerable patients - Availability of appropriate materials to set up equitable care - Knowledge of own patient populationInterdisciplinarity of the team
Other healthcare professionals and city and social services	- Limited availability for patients and GP practice staff - Low accessibility for vulnerable patients - Lack of communication	- Strong (existing) interprofessional collaborative networks with GP practice staff - Approachability for advising the GP practice staff - Clear agreements with the GP practice staff	- Limited availability for patients and GP practice staff - Low accessibility for vulnerable patients - Lack of communication	- Strong (existing) interprofessional collaborative networks - Availability to collaborate with the GP practice staff - Clear agreements with the GP practice staff
Government and professional organizations	- Rapidly changing or confusing guidelines on care provision - Unrealistic preconditions or guidelines for care provision - Fragmented organization	- Smooth spread of information about PHSMs and guidelines on care provision	/	/
Media	- Poor accessibility for vulnerable patients - Disseminated misinformation	- Broad reach among the general population	/	/

GP = general practitioner; PHSMs = public health and social measures.

**Table 2:** The characteristics of the participating GPs in this study.

Code GP	Gender	Work Experience	Migration Background	Location
Study population in Flanders				
GPF1	Male	15–20 y	No	Vlaams-Brabant
GPF2	Female	0–5 y	No	Oost-Vlaanderen
GPF3	Female	0–5 y	No	Oost-Vlaanderen
GPF4	Male	10–15 y	No	Oost-Vlaanderen
GPF5	Male	30–35 y	No	West-Vlaanderen
GPF6	Female	35–40 y	No	Oost-Vlaanderen
GPF7	Male	25–30 y	No	Oost-Vlaanderen
GPF8	Female	0–5 y	No	West-Vlaanderen
GPF9	Female	20–25 y	No	West-Vlaanderen
GPF10	Male	40–45 y	No	Oost-Vlaanderen
GPF11	Male	0–5 y	Yes	Oost-Vlaanderen
GPF12	Male	35–40 y	No	Oost-Vlaanderen
GPF13	Male	30–35 y	No	Oost-Vlaanderen
GPF14	Female	0–5 y	No	West-Vlaanderen
GPF15	Male	0–5 y	No	Limburg
GPF16	Male	35–40 y	No	Oost-Vlaanderen
GPF17	Female	35–40 y	No	Limburg
GPF18	Male	30–35 y	No	Oost-Vlaanderen
Study population in The Netherlands				
GPN1	Female	0–5 y	No	Zuid-Holland
GPN2	Female	35–40 y	No	Noord-Holland
GPN3	Female	0–5 y	Yes	Gelderland
GPN4	Male	10–15 y	No	Gelderland
GPN5	Female	15–20 y	No	Zuid-Holland
GPN6	Female	20–25 y	No	Noord-Brabant
GPN7	Female	25–30 y	No	Gelderland
GPN8	Male	5–10 y	Yes	Overijssel
GPN9	Male	0–5 y	No	Gelderland
GPN10	Female	35–40 y	No	Utrecht
GPN11	Female	25–30 y	No	Gelderland
GPN12	Male	5–10 y	Yes	Noord-Brabant
GPN13	Female	5–10 y	No	Noord-Holland
GPN14	Female	20–25 y	No	Noord-Holland
GPN15	Female	10–15 y	No	Utrecht
GPN16	Female	20–25 y	No	Utrecht

GP = general practitioner; y = years.

## Data Availability

All data were pseudonymized and securely stored on the server of Ghent University or Radboud University, respectively. The key to the unique codes to identify the participants was only accessible to the researchers E.V.P., T.v.L. and M.V.d.M. Reasonable request is required to access non-identifiable data by users who are external to the research teams involved. Access will be subject to a data transfer agreement and following approval from the principal investigators of this study.

## Supplementary Materials

The following supporting information can be downloaded at: https://www.mdpi.com/article/10.3390/healthcare11233009/s1, Supplementary Table S1: The consolidated criteria for reporting qualitative studies (COREQ checklist).
