# Supplementary material for: Barriers and Enablers Experienced by General Practitioners in Delivering Safe and Equitable Care during COVID-19: A Qualitative Investigation in Two Countries
Source: Healthcare (Basel). 2023 Nov 21;11(23):3009. doi: 10.3390/healthcare11233009 (PMC10706011; doi:10.3390/healthcare11233009)
Supplement: Supplementary file 1 [file healthcare-11-03009-s001.zip › healthcare-2684800-supplementary.pdf]

Supplementary Material

Supplementary Table S1. The consolidated criteria for reporting qualitative studies (COREQ checklist)

No. Item	Guide questions/description	Remarks	Reported on Page #
**Domain 1: Research team and reflexivity**			
Personal Characteristics			
1. Interviewer/facilitator	Which author/s conducted the interview or focus group?	K.V.R. conducted the in-depth interviews<br>As mentioned in the acknowledgement section, L.R. and A.V.D.D. also contributed to the data collection	16
2. Credentials	What were the researcher's credentials? E.g. PhD, MD	E.V.P: Msc<br>T.v.L, C.C., and S.W.: PhD<br>K.V.R. and M.V.d.M.: PhD, MD	1
3. Occupation	What was their occupation at the time of the study?	The researchers' occupations are as follows:<br>E.V.P., T.v.L., and S.W.: academic<br>M.V.d.M.: general practitioner and academic<br>C.C.: professional organization for general practitioners and academic	1
4. Gender	Was the researcher male or female?	All researchers were female	1
5. Experience and training	What experience or training did the researcher have?	All researchers have extended experience in qualitative research and/or quality of healthcare in general practice	2
Relationship with participants			
6. Relationship established	Was a relationship established prior to study commencement?	All GPs were invited via e-mail through the network of the Flemish and Dutch research teams. In the Netherlands, the participants did not have prior knowledge of the interviewer before the study. Participants received an information letter	2

		about the study, which included details about the research team, including contact information. The interviewer, prior to the interview, explained her background and the study's purpose.	
7. Participant knowledge of the interviewer	What did the participants know about the researcher? e.g. personal goals, reasons for doing the research	A few Flemish participants knew their interviewer prior the study through their engagement at the department of Public Health and Primary Care (Ghent University, BE). The remaining participants did not know any of the researchers, although they were all aware that the interview was conducted for research purposes.	2
8. Interviewer characteristics	What characteristics were reported about the interviewer/facilitator? e.g. Bias, assumptions, reasons and interests in the research topic	K.V.R. and L.R. have experience in qualitative research and were affiliated to the research unit 'Equity in Health Care' at the department of Public Health and Primary Care (Ghent University, BE). A.V.D.D. had a background in general practice and was a trainee at Radboud University Medical Centre (Nijmegen, NL).	1, 16

**Domain 2: study design**			
Theoretical framework			
9. Methodological orientation and Theory	What methodological orientation was stated to underpin the study? e.g. grounded theory, discourse analysis, ethnography, phenomenology, content analysis	Thematic analysis was applied in line with the six-step analysis procedure introduced by Braun and Clarke	3
Participant selection			

10. Sampling	How were participants selected? e.g. purposive, convenience, consecutive, snowball	Purposive sampling was employed via the networks of the research teams.	2
11. Method of approach	How were participants approached? e.g. face-to-face, telephone, mail, email	GPs were invited by e-mail to participate in the study.	
12. Sample size	How many participants were in the study?	18 Flemish and 16 Dutch GPs participated in the study.	2
13. Non-participation	How many people refused to participate or dropped out? Reasons?	None	n/a
Setting			
14. Setting of data collection	Where was the data collected? e.g. home, clinic, workplace	Due to the public health and social measures at that time, interviews occurred online in Flanders and over the telephone in the Netherlands.	3
15. Presence of non-participants	Was anyone else present besides the participants and researchers?	No	n/a
16. Description of sample	What are the important characteristics of the sample? e.g. demographic data, date	The important characteristics of the samples include gender, work experience, migration background, and location.	3
Data collection			
17. Interview guide	Were questions, prompts, guides provided by the authors? Was it pilot tested?	The interview guides were developed based on expert opinion, and are available on reasonable request for users external to the research team. No pilot testing took place.	3
18. Repeat interviews	Were repeat interviews carried out? If yes, how many?	No	3
19. Audio/visual recording	Did the research use audio or visual recording to collect the data?	Interviews were audio-visually recorded in Flanders and audio recorded in the Netherlands.	3

20. Field notes	Were field notes made during and/or after the interview or focus group?	No	n/a
21. Duration	What was the duration of the interviews or focus group?	In both regions, the interviews lasted approximately 60 minutes.	3
22. Data saturation	Was data saturation discussed?	Data saturation was discussed in the methods and discussion section.	4,15
23. Transcripts returned	Were transcripts returned to participants for comment and/or correction?	No	16
**Domain 3: analysis and findings**			
Data analysis			
24. Number of data coders	How many data coders coded the data?	Initially, researchers E.V.P. and K.V.R. collaboratively coded two interviews. They then collaborated to align on codes and emerging themes. M.V.d.M. applied the preliminary Flemish coding structure to Dutch interviews, refining it. Discussions between the Flemish (E.V.P.) and Dutch team (T.v.L., M.V.d.M.) resulted in a shared coding tree. E.V.P. subsequently reevaluated prior interviews using this shared framework for the remaining interviews.	3
25. Description of the coding tree	Did authors provide a description of the coding tree?	Table 1 shows the coding tree illustrating barriers and enablers for GPs in delivering safe and equitable care, linked to various stakeholders.	3-4
26. Derivation of themes	Were themes identified in advance or derived from the data?	The themes that were identified were derived from the data.	3

27. Software	What software, if applicable, was used to manage the data?	The Flemish transcripts were imported into NVivo release 1.6.1. and the Dutch transcripts into Atlas.ti.	3
28. Participant checking	Did participants provide feedback on the findings?	No, the participants did not provide any feedback on the findings.	n/a
Reporting			
29. Quotations presented	Were participant quotations presented to illustrate the themes/findings? Was each quotation identified? e.g. participant number	Yes, quotations were presented and identified by the unique code of the participant (number and country).	6-12
30. Data and findings consistent	Was there consistency between the data presented and the findings?	Yes, the data presented aligned consistently with the findings.	6-15
31. Clarity of major themes	Were major themes clearly presented in the findings?	Yes, major themes were clearly presented in the findings and discussion section.	6-15
32. Clarity of minor themes	Is there a description of diverse cases or discussion of minor themes?	Yes, diverse cases were presented thorough the results and discussion section.	6-15
